# Effect of (short-term) intravenous iron supplementation in iron-deficient non-anaemic cardiac surgical patients on perioperative outcome

**DOI:** 10.1186/s13741-025-00596-8

**Published:** 2025-10-13

**Authors:** Lea Valeska Blum, Nico Hipp, Vanessa Neef, Anatol Prinzing, Kai Zacharowski, Patrick Meybohm, Suma Choorapoikayil

**Affiliations:** 1 Department of Anaesthesiology, Intensive Care and Pain Therapy, University Hospital Frankfurt, Goethe University Frankfurt, Frankfurt, Germany; 2 Department of Cardiovascular Surgery, University Hospital Frankfurt, Goethe University Frankfurt, Frankfurt, Germany; 3 Department of Anaesthesiology, Intensive Care, Emergency and Pain Medicine, University Hospital Würzburg, Würzburg, Germany

**Keywords:** Iron deficiency, Outcome, Hospital

## Abstract

**Background:**

While intravenous iron improves outcomes in anaemic surgical patients, the impact of iron deficiency (ID) and its treatment in non-anaemic patients remains unclear.

**Methods:**

In this single-centre retrospective analysis, non-anaemic ID patients (age ≥ 18 years) undergoing major cardiac surgery at the University Hospital Frankfurt were included. Primary endpoints were red blood cell (RBC) transfusion rate and use of RBC units. Secondary endpoints were increase in haemoglobin levels and postoperative outcome (mortality, length of stay, mechanical ventilation, laboratory values). Patients were assigned to the following groups: No-Iron (no anaemia, ID, and no iron supplementation) and Iron (anaemia, ID, and iron supplementation).

**Results:**

A total of 3605 patients were screened, of whom 2345 were non-anaemic. Six hundred ninety-eight non-anaemic ID patients were included in the analysis, of whom 90 received intravenous iron supplementation. The overall RBC transfusion rate (43.6% [95% CI: 39.6–47.6] versus 50.0% [95% CI: 39.9–60.1]) and number of transfused blood units (2.0 [IQR: 1.0; 4.0] versus 2.0 [IQR: 1.0; 4.0]) were similar between patients of the No-Iron and Iron groups. Hospital length of stay, mortality, and postoperative complications were similar in both groups. When applying stricter cutoff values to define ID (ferritin < 30 μg/l), a trend toward reduced transfusion rates was observed: total RBC transfusion rate was 50.0% (95% CI: 34.9–65.2) in the No-Iron group and 42.9% (95% CI: 26.8–60.5) in the Iron group. In the case of short-term (1 day prior to surgery) iron supplementation, RBC unit utilisation and postoperative outcomes were comparable between the two groups.

**Conclusion:**

In non-anaemic cardiac surgery patients, (short-term) preoperative intravenous iron supplementation showed no significant impact on RBC transfusion rate, haemoglobin levels, or postoperative outcomes. However, a stricter definition of ID revealed a trend toward reduced transfusion rates.

**Supplementary Information:**

The online version contains supplementary material available at 10.1186/s13741-025-00596-8.

## Introduction

Anaemia is a well-established risk factor for outcome in patients undergoing major surgery. The prevalence of preoperative anaemia varies between 10.5% and up to 47.9% (Muñoz et al. 2015). Iron deficiency (ID) is the leading cause of preoperative anaemia. In anaemic patients undergoing surgery, intravenous iron (IV) supplementation has been associated with an increase in haemoglobin (Hb) and improved postoperative outcomes including reduced red blood cell (RBC) transfusions and hospital length of stay (LOS) (Triphaus et al. 2021; Hung et al. 2024; Spahn et al. 2019). While the deleterious effects of ID anaemia on postoperative outcomes have been extensively studied, the impact of ID in the absence of anaemia remains poorly understood. Rössler et al. compared the postoperative outcomes of non-anaemic and anaemic patients with and without ID undergoing cardiac surgery. A preoperative serum ferritin level of < 100 μg/l was associated with an increased risk of mortality, rising from 2 to 5% in patients without anaemia and from 4 to 14% in those with anaemia. Similarly, ID was associated with an increase in major adverse cardiac and cerebrovascular events from 5 to 8% without and from 5 to 19% with anaemia (Rössler et al. 2020). It is noteworthy that (postoperative) RBC transfusion requirements may be higher in patients with ID compared to those without (Horwood et al. 2024; Peri et al. 2024). Most studies have assessed the impact of IV iron supplementation in ID anaemic patients due to the association between preoperative anaemia and postoperative morbidity. To date, the role of iron supplementation in non-anaemic iron-deficient surgical patients remains unclear. Since iron is essential for Hb production, iron-deficient patients are unable to adequately compensate for surgical blood loss in the postoperative period, leading to an increased need for transfusion. In this pilot study, we investigated the impact of IV iron supplementation on perioperative outcomes in iron-deficient non-anaemic patients undergoing major cardiac surgery. The primary endpoint was the change in RBC transfusion requirements, and the secondary endpoints were the effects of IV iron supplementation on Hb levels and postoperative outcomes, including mortality and LOS.

## Material and methods

This study is a single-centre retrospective analysis of data from a multicentre observational epidemiological trial focusing on the implementation of Patient Blood Management (PBM) in surgical patients (Meybohm et al. 2023). The study protocol was approved by the ethics committee of the University Hospital Frankfurt (Ref. 318/17) and the requirement for written informed consent by patients was waived.

### Patients and procedures

Patients (age ≥ 18 years) scheduled for major cardiac surgery with a ≥ 10% probability of RBC transfusion or ≥ 500 ml blood loss (Meybohm et al. 2020), and screened for preoperative anaemia and ID at the anaemia walk-in clinic (Supplemental Table 1) from November 2017 to May 2023, were included in our analysis. 

In 2017, the clinic’s screening protocol was revised to include assessment of iron status in non-anaemic patients. On a daily basis, the anaemia nurse reviewed the laboratory profiles of patients scheduled for major surgery according to a defined patient pathway (Anemia Algorithm V04 (2025)). If blood was collected during the pre-examination, ferritin and transferrin saturation were analysed. If no blood samples were taken, patients were invited to the anaemia walk-in clinic for diagnosis (Triphaus et al. 2021).

Patients were excluded from analysis in case of planned reoperation or transcatheter aortic valve implantation, incomplete data, Jehovah’s witness, or massive transfusion (> 10 transfused RBCs within 24 hours). The use of cell salavage was based on local blood conservation strategy guidelines suggesting its use when the anticipated blood loss exceeds 400 ml. In surgeries involving cardiopulmonary bypass, a substantial proportion of blood is returned directly to the patient’s circulation via the bypass circuit, resulting in a comparatively low cell salvage yield in these cases.

All analysed data were extracted from the electronic hospital information system.

### Endpoints

Primary endpoints were transfusion rate and use of RBC units during hospital stay. Secondary endpoints were Hb increase and postoperative complications (renal replacement therapy, cerebral ischaemic events, antibiotic therapy, sepsis, reoperation, and the duration of mechanical ventilation). Antibiotic therapy was defined as the requirement for an additional antibiotic, beyond perioperative antibiotic prophylaxis, for a confirmed or suspected infection.

### Classification of anaemia and iron deficiency

According to the World Health Organization (WHO) anaemia is defined as an Hb concentration < 12 g/dl in women and < 13 g/dl in men. Iron deficiency was diagnosed by laboratory results according to Munoz et al. (2017) and Anker et al. (2009) and defined as a serum ferritin < 100 μg/l or ferritin < 300 μg/l with transferrin saturation < 20%. In addition, a full medical history of the patient was considered during diagnosis. Accordingly, patients were assigned to the following groups: No-Iron (no anaemia, ID, and no iron supplementation) and Iron (no anaemia, ID, and iron supplementation). Other common causes of anaemia such as anaemia of inflammation, anaemia of chronic renal disease, and folate or vitamin B12 deficiency were not primarily addressed.

### Iron supplementation

Patients with ID received IV iron (ferric carboxymaltose, 50 mg/ml; Vifor, Saint Gallen, Switzerland or Monofer 100 mg/ml; Pharmacosmos, Holbaek, Denmark) administered at a dose of either 500 mg in 100 ml saline over 15 min or 1000 mg in 250 ml saline over 30 min, depending on laboratory results (Hb levels, body mass index, ferritin level, transferrin saturation, and transferrin receptor levels). Contraindications included a history of hypersensitivity reactions, acute infection requiring antibiotics, and conditions of iron overload or impaired iron metabolism (e.g. haemochromatosis). During and after the IV iron infusion, patients were actively questioned about any discomfort to ensure prompt care in the event of an adverse reaction. Vital signs and occurrence of adverse events (itching, flush, urticarial, angioedema, nausea, seizures, vomiting, defaecation, rhinorrhoea, hoarseness, coughing, dyspnoea, apnoea, tachycardia, hypotension, arrhythmia, shock, circulatory arrest, and headache) were monitored during the infusion and for an additional 10 to 15 min before discharge.

### Red blood cell transfusion

RBC transfusions were performed in accordance with the ‘Cross-sectional Guidelines for Therapy with Blood Components and Plasma Derivatives’ (Bundesärztekammer 2021; 2009).

### Statistical analysis

Due to the retrospective design of the study, a priori power analysis was not performed, and the sample size was determined based on the available data. Descriptive variables were analysed using means with 95% confidence interval (CI) and medians with interquartile ranges (IQRs) (P25%; P75%), as well as counts and percentages. Statistical significance was considered when *p* < 0.05. Data were checked for normal distribution using the Shapiro–Wilk test. For non-normally distributed data, differences between groups were assessed using the Wilcoxon rank sum test with continuity correction. For mean comparisons, the Welch two-sample *t*-test was additionally applied. For categorial variables, Pearson’s chi-squared test with Yates’ continuity correction was used. To calculate the Hb increment after IV iron supplementation, the first Hb level measured at the anaemia walk-in clinic or during pre-examination and the last Hb level within 24 h before surgery were used (Wilcoxon signed-rank test). To compare outcome data of patients treated 1 day before surgery, propensity matching was performed based on gender, age, body mass index, type of surgery, preoperative Hb level, ferritin, transferrin saturation, mean corpuscular haemoglobin, and mean corpuscular volume, gender, vitamin K antagonist/DOAC, and COPD using the nearest neighbour method (ratio = 1) within the MatchIt package in R. Patients of the Iron group with short-term iron supplementation (1 day prior to surgery) were compared to patients of the No-Iron group. The quality of matching was assessed by comparing covariate distributions before and after matching. Given the ongoing debate on diagnostic thresholds for ID, we applied a more restrictive ferritin cutoff (ferritin < 30 μg/l) to explore whether this better identifies patients likely to show a Hb response to treatment, and performed a post hoc analysis to assess RBC transfusion in this subgroup. To explore differences in transfusion rates by treatment group and sex, we stratified the dataset by sex and calculated transfusion rates within each group (Iron vs. No-Iron). For each subgroup, the number of individuals, the number of transfusions, and the proportion transfused were computed using the dplyr package in R. Differences were assessed using Fisher’s exact tests. All analyses and graphical illustrations were performed using R software (version 4.2.2 (2022-10-31)) and Microsoft Excel (2016).

## Results

**Fig. 1:**
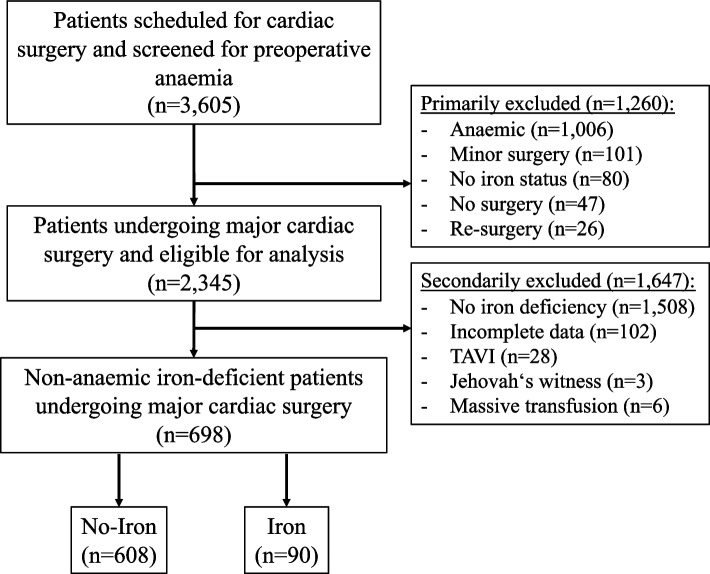
Flow chart. In total, 80 patients were not screened for iron deficiency due to organisational issues, including a short time interval between screening and surgery, patients’ non-compliance with making appointments, and the illness of the anaemia nurse. TAVI = transcatheter aortic valve implantation

In total, 3605 patients were scheduled for cardiac surgery, of which 2345 (65.0%) were non-anaemic. Among the non-anaemic patients, 698 (29.8%) patients were iron-deficient and included in the analysis (Fig. 1). Out of the 698 iron-deficient non-anaemic patients, 90 received IV iron supplementation (Iron) and 608 patients received no supplementation (No-Iron) before surgery. Mean age was 64.6 (95% CI: 63.7–65.5) and 63.9 (95% CI: 61.4–66.4) in the No-Iron and Iron group, respectively. Female sex was significant lower in the No-Iron group (36.0% [95% CI: 32.0–40.0]) and 50.0% [95% CI: 40.0–60.0], *p* < 0.01). The prevalence of comorbidities and medications was similar between both groups except for chronic obstructive pulmonary disease and use of vitamin K antagonist or DOACs (Table 1).

**Table 1:** Patient characteristics

	**No-Iron** ***N* = 608**	**Iron** ***N* = 90**	*p* value*
Age (years)	64.6 (95% CI: 63.7–65.5)	63.9 (95% CI: 61.4–66.4)	0.82
Sex (female)	36.0% (95% CI: 32.0–40.0)	50.0% (95% CI: 40.0–60.0)	0.01
BMI (kg/m^2^)	27.2 (95% CI: 26.8–27.7)	28.4 (95% CI: 27.2–29.6)	0.04
Euroscore	3.5% (95% CI: 3.1–4.0)	4.0% (95% CI: 2.7–5.3)	0.37
ASA			
Score II	0.5% (95% CI: 0.0–0.0)	2.2% (95% CI: 0.0–0.1)	0.25
Score III	27.5% (95% CI: 0.2–0.3)	30.0% (95% CI: 0.2–0.4)	0.71
Score IV	71.6% (95% CI: 67.8–75.1)	67.4% (95% CI: 55.9–76.0)	0.41
Score V	0.5% (95% CI: 0.1–1.6)	0.0% (95% CI: 0.0–5.1)	1.00
LVEF preoperative	58.0% (95% CI: 57.1–58.9)	55.5 (95% CI: 52.7–58.4)	0.11
≤ 40	12.0% (95% CI: 9.6–14.9)	14.4% (95% CI: 8.2–23.8)	0.63
41–49	7.6% (95% CI:5.7–10.0)	7.8% (95% CI: 3.5–15.9)	1.00
≥ 50	80.4% (95% CI: 77.0–83.5)	77.8% (95% CI: 67.6–85.6)	0.66
**Comorbidities/medication**			
Obesity	24.8% (95% CI: 21.5–28.5)	34.4% (95% CI: 25.0–45.3)	0.07
Essential hypertension	75.0% (95% CI: 71.3–78.4)	81.1% (95% CI: 71.2–88.3)	0.26
Active smoker	23.0% (95% CI: 19.8–26.6)	32.9% (95% CI: 22.0–41.9)	0.12
Hypercholesteremia	62.0% (95% CI: 58.0–65.9)	54.4% (95% CI: 43.6–64.9)	0.21
Diabetes mellitus	28.1% (95% CI: 24.6–31.9)	31.1% (95% CI: 22.0–41.9)	0.65
NIDDM	17.4% (95% CI: 14.6–20.7)	21.1% (95% CI: 13.5–31.2)	0.48
IDDM	10.7% (95% CI: 8.4–13.5)	10.0% (95% CI: 5.0–18.6)	0.98
Stroke/TIA	8.2% (95% CI: 6.2–10.8)	5.6% (95% CI: 2.1–13.1)	0.50
COPD	6.9% (95% CI: 5.1–9.3)	15.6% (95% CI: 9.1–25.1)	0.009
CKD	13.2% (95% CI: 10.6–16.2)	12.2% (95% CI: 6.6–21.2)	0.93
Dialysis	0.0% (95% CI: 0.0–0.8)	1.2% (95% CI: 0.1–6.9)	
Platelet aggregation inhibitor	57.9% (95% CI: 53.9–61.8)	50.00% (95% CI: 39.9–60.1)	0.19
Vitamin K antagonist/DOAC	16.0% (95% CI: 13.2–19.2)	29.8% (95% CI: 19.1–38.4)	0.009

*BMI* Body mass index, *ASA* American Society of Anesthesiologists, *LVEF* Left ventricular ejection fraction, *NIDDM* Non-insulin-dependent diabetes mellitus, *IDDM* Insulin-dependent diabetes mellitus, *TIA* Transient ischaemic attack, *COPD* Chronic obstructive pulmonary disease, *CKD* Chronic kidney disease, *DOAC* Direct oral anticoagulants

^*^All analyses were performed using Wilcoxon rank sum test, except for categorial variables, which were analysed using Pearson’s chi-squared test

The first measured Hb level in the anaemia walk-clinic was 14.1 (95% CI: 14.0–14.2) g/dl and 13.5 (95% CI: 13.3–13.7) g/dl in the No-Iron and Iron group (*p* < 0.001), respectively (Table 2). When stratified by sex, the mean Hb level was slightly lower in the Iron group compared to the No-Iron group for both females and males. Among females, the mean Hb was 13.0 g/dl (95% CI: 12.8–13.3) in the Iron group compared to 13.4 g/dl (95% CI: 13.3–13.5) in the No-Iron group (*p* = 0.011). Among males, the mean Hb was 13.9 g/dl (95% CI: 13.7–14.2) in the Iron group compared to 14.5 g/dl (95% CI: 14.4–14.6) in the No-Iron group (*p* < 0.001).

**Table 2:** Haemoglobin values and blood transfusion

	**No-Iron** ***N* = 608**	**Iron** ***N* = 90**	***p* value**
First measured Hb (g/dl)	14.1 (95% CI: 14.0–14.2)	13.5 (95% CI: 13.3–13.7)^a^	< 0.001
Hb at admission ICU (g/dl)	9.9 (95% CI: 9.8–10.0)	9.9 (95% CI: 9.6–10.1)	0.74
Hb at discharge (g/dl)	10.1 (95% CI: 10.0–10.2)	10.0 (95% CI: 9.7–10.3)	0.30
RBC transfusion rate			
Total	43.6% (95% CI: 39.6–47.6)	50.0% (95% CI: 39.9–60.1)	0.18
Intraoperative (%)	24.3% (95% CI: 21.0–28.0)	31.1% (95% CI: 22.0–41.9)	0.21
Postoperative (%)	31.4% (95% CI: 27.8–35.3)	33.3% (95% CI: 24.0–44.2)	0.81
RBC/Pat. total (units)	2.0 (IQR: 1.0; 4.0)	2.0 (IQR: 1.0; 4.0)	0.42
RBC/Pat. intraoperative (units)	1.0 (IQR: 1.0; 2.0)	1.0 (IQR: 1.0; 2.0)	0.53
RBC/Pat. postoperative (units)	2.0 (IQR: 1.0; 3.0)	2.0 (IQR: 1.0; 3.0)	0.78
Use of cell salvage	28.6% (95%CI: 25.1–32.4)	20% (95%CI: 12.6–30.0)	0.11
Retransfusion cell salvaged blood (ml)	407.2 (95% CI:361.5–453.0)	626.1 (95% CI: 411.3–840.8)	0.02
Retransfusion CPB (ml)	458.3 (95% CI: 441.7–474.9)	497.4 (95% CI: 456.1–538.7)	0.02

*RBC* Red blood cell, *Hb* Haemoglobin, *Pat.* Patient, *CPB* Cardiopulmonary bypass

^a^Hb level before iron supplementation

In the Iron group, IV iron was administrated a median of 1 (IQR: 1; 2) day before surgery (Supplemental Fig. 1A). No serious adverse events occurred during and after iron administration. In the Iron group, Hb changes were observed, ranging from −0.8 to 0.9 g/dl (Supplemental Fig. 1B) (*p* = 0.4212).

Compared to No-Iron, patients of the Iron group had a significantly higher retransfused blood volume (Table 2).

The overall RBC transfusion rate (43.6% [95% CI: 39.6–47.6] versus 50.0% [95% CI: 39.9–60.1]) and number of transfused blood units (2.0 [IQR: 1.0; 4.0] versus 2.0 [IQR: 1.0; 4.0]) were similar between both groups (Table 2, Supplemental Fig. 2 A and Supplemental Fig. 3). When stratified by sex, the odds of RBC transfusion among females were higher in the Iron group compared to the No-Iron group (OR of 1.85 [95% CI: 0.88–4.06; *p* = 0.096]). Among males, the odds were lower in the Iron group (OR 0.72 [95% CI: 0.34–1.47]; *p* = 0.411).

Hospital LOS, mortality, and postoperative complications including mechanical ventilation were similar in both groups (Table 3 and Supplemental Fig. 4). In addition, we found no significant differences in kidney function or phosphate values (Table 4). Compared to the No-Iron group, patients of the Iron group had higher Il-6 (pg/ml) values at ICU admission (743.2 [95% CI: 554.7–931.7] vs. 1019.5 [95% CI: 485.7–1553.2], *p* = 0.04) (Table 4). At discharge from ICU, Il-6 values (pg/ml) were similar in both groups (207.2 [95% CI: 143.7–270.8] vs. 315.2 [95% CI: 105.6–524.8]).

**Table 3:** Postoperative outcome

	**No-Iron** ***N* = 608**	**Iron** ***N* = 90**	***p* value**
Hospital LOS (days)	10.4 (95% CI: 9.8–10.9)	11.3 (95% CI: 9.6–12.9)	0.28
ICU LOS (days)	2.3 (95% CI: 2.0–2.5)	2.6 (95% CI: 1.8–3.5)	0.42
Mortality by any cause	3.0% (95% CI: 1.8–4.7)	4.4% (95% CI: 1.4–11.6)	0.67
**Complications (%)**			
LVEF postoperative	57.4% (95% CI: 56.6–58.3)	56.8% (95% CI: 54.1–59.4)	0.61
≤ 40	8.2% (95% CI: 6.2–10.8)	13.3% (95% CI: 7.4–22.5)	0.16
41–49	10.2% (95% CI: 8.0–13.0)	12.2% (95% CI: 6.6–21.2)	0.69
≥ 50	81.6% (95% CI: 78.2–84.5)	74.4% (95% CI: 64.0–82.8)	0.15
Dialysis	5.1% (95% CI: 3.6–7.2)	7.8% (95% CI: 3.5–15.9)	0.42
Cerebral ischemic event	3.0% (95% CI: 1.8–4.7)	0.0% (95% CI: 0.0–5.1)	0.19
Antibiotic therapy	20.2% (95% CI: 17.2–23.7)	20.0% (95% CI: 12.6–30.0)	1.00
Re-surgery	6.7% (95% CI: 4.9–9.1)	12.2% (95% CI: 6.6–21.2)	0.10
Sepsis	1.6% (95% CI: 0.8–3.1)	3.3% (95% CI: 0.9–10.1)	0.49
Duration of mechanical ventilation (h)	57.9 (95% CI: 43.1–72.7)	60.8 (95% CI: 23.9–97.7)	0.79

*LOS* Length of stay, *ICU* Intensive care unit, *LVEF* Left ventricular ejection fraction

**Table 4:** Laboratory profile

	**No-Iron** ***N* = 608**	**Iron** ***N* = 90**	***p* value**
**Preoperative**			
Creatinine (mg/dl)	1.0 (95% CI: 1.0–1.0)	1.1 (95% CI: 0.9–1.3)	0.90
eGFR to CKD-EPI (ml/min/1.73 m^2^)	77.1 (95% CI: 75.6–78.6)	74.7 (95% CI: 70.2–79.2)	0.29
Urea (mg/dl)	37.8 (95% CI: 36.7–39.0)	36.6 (95% CI: 33.4–39.8)	0.22
Interleukin-6 (pg/ml)	5.7 (95% CI: 5.0–6.5)	5.6 (95% CI: 4.3–6.8)	0.24
**Postoperative**^**a**^			
Phosphate (mg/dl)	3.4 (95% CI: 3.4–3.5)	3.4 (95% CI: 3.2–3.6)	0.21
Creatinine (mg/dl)	0.9 (95% CI: 0.9–1.0)	1.0 (95% CI: 0.9–1.2)	0.70
eGFR to CKD-EPI (ml/min/1.73 m^2^)	79.2 (95% CI: 77.7–80.7)	76.7 (95% CI: 72.4–81.0)	0.47
Urea (mg/dl)	32.6 (95% CI: 31.2–34.0)	31.7 (95% CI: 28.8–34.6)	0.21
Interleukin-6 (pg/ml)	743.2 (95% CI: 554.7–931.7)	1019.5 (95% CI: 485.7–1553.2)	0.04
**Discharge ICU**			
Phosphate (mg/dl)	3.8 (95% CI: 3.7–3.9)	3.8 (95% CI: 3.5–4.0)	0.96
Creatinine (mg/dl)	1.2 (95% CI: 0.9–1.4)	1.1 (95% CI: 0.9–1.2)	0.91
eGFR to CKD-EPI (ml/min/1.73 m^2^)	78.0 (95% CI: 76.1–79.9)	75.3 (95% CI: 70.0–80.7)	0.45
Urea (mg/dl)	40.7 (95% CI: 38.8–42.6)	38.6 (95% CI: 34.2–42.9)	0.45
Interleukin-6 (pg/ml)	207.2 (95% CI: 143.7–270.8)	315.2 (95% CI: 105.6–524.8)	0.75

*eGFR* Estimated glomerular filtration rate, *CKD-EPI* Chronic kidney disease epidemiology collaboration, *Hb* Haemoglobin, *ICU* Intensive care unit

^a^First measured value after surgery

### Effect of short-term intravenous iron supplementation

An analysis of propensity score-matched patients was performed to assess the effect of short-term iron supplementation on postoperative outcome. Intra- and postoperative transfusion rate and number of transfused RBC units were similar in both groups (Supplemental Table 2 and Supplemental Fig. 2B). No significant difference was detected in the postoperative outcome (Supplemental Table 3 and Supplemental Table 4).

### Red blood cell transfusion in patients with ferritin < 30 µg/l

In total, 38 patients of the No-Iron and 35 patients of the Iron group had ferritin values < 30 µg/l. The total RBC transfusion rate was 50.0% (95% CI: 34.9–65.2) in the No-Iron group and 42.9% (95% CI: 26.8–60.5) in the Iron group (Supplemental Table 5 and Supplemental Fig. 2 C).

## Discussion

The prevalence of ID in non-anaemic patients undergoing major surgery was high in our analysis, reaching 29.8%. Intravenous iron supplementation in ID patients with preoperative Hb values above 12-13 g/dl was not associated with improved postoperative outcomes compared to untreated non-anaemic ID patients.

Excessive perioperative blood loss often necessitates a blood transfusion. Despite efforts to avoid blood transfusion, it remains one of the most common treatments for perioperative anaemia. Iron deficiency is a leading cause of preoperative anaemia (Muñoz et al. 2017; Piednoir et al. 2011), and preoperative IV iron supplementation has been associated with increased Hb level, reduced transfusion rates, and shorter hospital LOS (Hung et al. 2024; Corsi et al. 2023). The effect of IV iron supplementation in non-anaemic but iron-deficient patients remains insufficiently established. However, iron-deficient non-anaemic patients who experience excessive blood loss may be unable to produce erythrocytes due to depleted iron stores.

A growing number of cardiac surgical patients underwent surgery within a few days of their first consultation at our hospital. Therefore, the majority of the analysed patients received IV iron supplementation 1 day before surgery. We observed similar RBC transfusion rate and number of transfused RBC units between treated and non-treated ID patients. However, it is noteworthy to mention that pre-transfusion Hb values were not assessed, with transfusion thresholds potentially varying according to the clinical context of each patient. Furthermore, hospital LOS, mortality, and postoperative complications including mechanical ventilation were similar in both groups. Given that erythrocyte maturation requires up to 6 days, a measurable increase in Hb levels would not be expected until at least several days after iron supplementation. Song and colleagues showed that a rise in reticulocytes and Hb was not noted until postoperative day 6 (Song et al. 2022).

In our matched-pair analysis, we found no significant differences in RBC transfusion rate or postoperative outcome. It is noteworthy that RBC transfusion is influenced by intraoperative decisions, which has not been addressed in the analysis. In addition, the complexity of the surgical procedures included in this study may have influenced the observed transfusion requirements. Patients undergoing combined procedures such as coronary artery bypass graft (CABG) and valve surgery or multivalve interventions are likely to experience longer operative times, greater blood loss, and higher perioperative risk compared to those undergoing isolated valve or CABG procedures. Aortic surgery, known for its technical difficulty and potential for significant bleeding, also adds to the heterogeneity of surgical complexity. Due to sample size limitations, outcomes by surgical type could not be stratified in the analysis. 

Interestingly, patients with ferritin values < 30 µg/l who received IV iron supplementation had a lower RBC transfusion rate (42.9% (95% CI: 26.8–60.5)) compared to non-treated patients (50.0% (95% CI: 34.9–65.2)), especially in the postoperative period (25.7% [95% CI: 13.1–43.6] vs. 39.5% [95% CI: 24.5–56.6]), although this difference did not reach statistical significance (*p* = 0.71).

Taking a pragmatic approach, Spahn et al. implemented a treatment bundle comprising a short-term administration of erythropoietin, IV iron, vitamin B12, and folate in patients with anaemia or ID 1 to 3 days prior to cardiac surgery. This multimodal intervention was associated with a decreased need for RBC transfusions within the first week postoperatively (Spahn et al. 2019). However, the benefit of IV iron supplementation in non-anaemic patients is controversially discussed. Recently, Friedman et al., analysed outcomes in 194 randomised patients undergoing CABG or valve surgery, 99 of whom received IV iron supplementation. Compared to the placebo group, IV iron supplemented patients had significant lower RBC transfusion (0.3 versus 1.6) by postoperative day 4 (Friedman et al. 2023). However, a key distinction between our study and Friedman’s is that our cohort comprises high-risk patients undergoing a broader range of cardiac surgical procedures.

In addition to the controversial discussion of the effectiveness of IV iron supplementation, the detrimental impact of ID is also emphasized. For example, Miles and colleagues showed that in non-anaemic patients undergoing elective cardiac surgery, presence of ID was not associated with increased duration of intensive care, hospital LOS, incidence of re-admission, requirement for RBC transfusion, or occurrence of postoperative complication (Miles et al. 2022). Kim et al. found no significant difference in days alive and out of hospital at postoperative day 90 between patients undergoing valve heart surgery with and without non-anaemic ID after adjustment for perioperative confounding factors (Kim et al. 2022). Anker et al. showed that supplementation of IV iron in patients with chronic heart failure and ID with or without anaemia improved symptoms, functional capacity, and the quality of life whereas mortality rate was not decreased (Anker et al. 2009).

It is important to address that the regulation of iron concentration is crucial to prevent toxicity (Ghafourian et al. 2020). When iron levels exceed the binding capacity of transferrin, the non-transferrin-bound iron can promote reactive oxygen species formation, leading to oxidative stress and potential organ dysfunction. Especially vasoplegia, when resulting in an overproduction of nitric oxide, can lead to further complications, particularly in patients with impaired left ventricular ejection fraction (LVEF) (van Vessem et al. 2017). In the heart, excessive iron accumulation has been linked to oxidative damage, disruptions in cardiac electrical activity, and fibrosis development. Conversely, ID has been associated with acute coronary syndrome, idiopathic pulmonary essential hypertension, cyanotic congenital heart disease, and worsening heart failure symptoms (Murphy and Oudit, 2010; Cacoub et al. 2022). Furthermore, ID is associated with reduced aerobic performance and physical condition. It is important to note that ID predispose to hematinic deficiencies and may delay the recovery from postoperative anaemia.

This study has several limitations. First, the cutoff values to diagnose ID have been rigorously debated in the last decades. The WHO recommends a ferritin cutoff value of < 15 µg/l in healthy individuals and < 70 µg/l in individuals with infection or inflammation for the diagnosis of ID (WHO 2020). In patients with chronic kidney disease or heart failure, ferritin values of up to 300 µg/l and transferrin saturation below 20% are also considered indicative for ID (Muñoz et al. 2017). Brautaset Englund and colleagues revealed that IV iron supplementation only improved peak oxygen consumption and replenished iron stores in heart transplant recipients with a ferritin level < 30 µg/l, but not in patients with ferritin level < 100 µg/l (Brautaset Englund et al. 2021). In addition, the greater relevance of transferrin saturation compared to ferritin as a diagnostic marker has been a topic of discussion in the last decade. Several studies have raised concerns about value of ferritin in the assessment of ID (Martens et al. 2023; Grote Beverborg et al. 2018). Therefore, the use of a more stringent cutoff value for diagnosing ID in a larger number of patients may have led to different outcomes. Second, because treatment allocation was not randomised but determined by individual clinical assessment, including patient condition and contraindications, the potential for selection bias cannot be excluded. In addition, the treatment group (Iron) comprised only 13% of the cohort, which introduce inherent selection bias. Patients receiving IV iron supplementation were not randomly assigned. This imbalance may limit statistical power and increase risk of type II error. Therefore, we cannot exclude the possibility that a larger, more balanced sample or a randomised allocation might have yielded different results. Furthermore, family-wise error correction for multiple outcomes was not applied. Third, the majority of patients received iron supplementation shortly before surgery. Elevated levels of pro-inflammatory cytokines after surgery often lead to the upregulation of hepcidin, a hormone that reduces the availability of iron for erythropoiesis (Wang and Babitt, 2016; Wittkamp et al. 2018). Therefore, the impact on Hb levels may only become evident after hospital discharge. Assessing the effect of IV iron supplementation in the post-discharge period would be an interesting target for future research. Fourth, the used WHO-recommended Hb threshold of 12 g/dl for diagnosing anaemia in women has been rigorously debated, with increasing emphasis on aligning it with the threshold used for men. Fifth, no information was available about oral iron therapy, which might have improved outcome data in patients of the No-Iron group. Finally, the Hb content of a single RBC unit can vary considerably depending on donor characteristics, processing methods, and storage conditions (Jain et al. 2022; Agnihotri et al. 2014). We did not record the Hb content of transfused units in our study, which may have influenced the observed transfusion outcomes.

## Conclusion

In non-anaemic cardiac surgical patients, (short-term) preoperative iron supplementation was not associated with significantly improved perioperative outcome, including Hb levels or reduced RBC transfusion requirements, compared with untreated non-anaemic ID patients. However, a stricter definition of ID in our non-anaemic patients revealed a trend toward reduced transfusion rates.

## Supplementary Information

Additional file 1: Supplemental Fig. 1. Time of iron supplementation before surgery. Supplemental Fig. 2. Utilisation of red blood cell units. RBC = red blood cell. Supplemental Fig. 3. Utilisation of red blood cell units per patient. RBC = red blood cell. Supplemental Fig. 4. Postoperative mechanical ventilation. Supplemental Table 1. Types of surgery included in analysis. CABG = coronary artery bypass graft. Supplemental Table 2. Utilisation of blood products and haemoglobin values in matched patients. RBC = red blood cell, CABG = coronary artery bypass graft, Hb = haemoglobin, *Hb level before iron supplementation, **Hb level after iron supplementation. Supplemental Table 3. Postoperative outcome in matched patients. LOS = length of stay, ICU = intensive care unit, LVEF = left ventricular ejection fraction. Supplemental Table 4. Laboratory profile in matched patients. eGFR = estimated glomerular filtration rate, CKD-EPI = chronic kidney disease epidemiology collaboration, Hb = haemoglobin, ICU = intensive care unit, *first measured value after surgery. Supplemental Table 5. Postoperative outcome in patients with ferritin < 30 µg/l. RBC = red blood cell, Hb = haemoglobin, Pat. = patient, CABG = coronary artery bypass graft, *Hb level before iron supplementation.

## Data Availability

The datasets used and/or analysed during the current study are available from the corresponding author on reasonable request.

## Abbreviations

CABG: Coronary artery bypass graft
CI: Confidence interval
DOAC: Direct oral anticoagulants
ICU: Intensive care unit
ID: Iron deficiency
IV: Intravenous iron
IQR: Inter-quartile range
Hb: Haemoglobin
LOS: Hospital length of stay
LVEF: Left ventricular ejection fraction
RBC: Red blood cell
WHO: World Health Organization

## References

[CR1] Agnihotri N, Pal L, Thakur M, Kumar P. The need to label red blood cell units with their haemoglobin content: a single centre study on haemoglobin variations due to donor-related factors. Blood Transfus. 2014;12(4):520–6. 24960649 10.2450/2014.0231-13 PMC4212032

[CR2] Anemia Algorithm V04. 2025. Available from: https://www.patientbloodmanagement.de/wp-content/uploads/2021/04/202103Anaemiemanagement_ENG.png.

[CR3] Anker SD, Comin Colet J, Filippatos G, Willenheimer R, Dickstein K, Drexler H, et al. Ferric carboxymaltose in patients with heart failure and iron deficiency. N Engl J Med. 2009;361(25):2436–48. 19920054 10.1056/NEJMoa0908355

[CR4] Brautaset Englund KV, Østby CM, Rolid K, Gude E, Andreassen AK, Gullestad L, et al. Intravenous iron supplement for iron deficiency in cardiac transplant recipients (IronIC): a randomized clinical trial. J Heart Lung Transplant off Publ Int Soc Heart Transplant. 2021;40(5):359–67. 10.1016/j.healun.2021.01.1390 33612360

[CR5] Bundesärztekammer, (ed). Querschnitts-Leitlinien zur Therapie mit Blutkomponenten und Plasmaderivaten. Köln: Deutscher Ärzteverlag; 2009 p 272 . Available from:https://www.bundesaerztekammer.de/fileadmin/user_upload/_old-files/downloads/QLL_Haemotherapie_2014.pdf.

[CR6] Bundesärztekammer, (ed). Querschnitts-Leitlinien zur Therapie mit Blutkomponenten und Plasmaderivaten: Gesamtnovelle; 2020. Deutscher Ärzteverlag; 2021. Available from:https://www.beck-elibrary.de/index.php?. 10.47420/9783769137309. Cited 2025 June 11.

[CR7] Cacoub P, Choukroun G, Cohen-Solal A, Luporsi E, Peyrin-Biroulet L, Peoc’h K, et al. Iron deficiency screening is a key issue in chronic inflammatory diseases: a call to action. J Intern Med. 2022;292(4):542–56. 10.1111/joim.13503 PMC9544998 35466452

[CR8] Corsi F, Pasquini A, Guerrera M, Bevilacqua F, Taccheri T, Antoniucci ME, et al. Single shot of intravenous iron in cardiac surgery: the ICARUS study. J Clin Anesth. 2023;84:111009. 36401886 10.1016/j.jclinane.2022.111009

[CR9] Friedman T, Dann EJ, Bitton-Worms K, Makhoul M, Glam R, Weis A, et al. Intravenous iron administration before cardiac surgery reduces red blood cell transfusion in patients without anaemia. Br J Anaesth. 2023;131(6):981–8. 37838604 10.1016/j.bja.2023.09.007

[CR10] Ghafourian K, Shapiro JS, Goodman L, Ardehali H. Iron and heart failure: diagnosis, therapies, and future directions. JACC Basic Transl Sci. 2020;5(3):300–13. 32215351 10.1016/j.jacbts.2019.08.009 PMC7091506

[CR11] Grote Beverborg N, Klip IjT, Meijers WC, Voors AA, Vegter EL, van der Wal HH, et al. Definition of iron deficiency based on the gold standard of bone marrow iron staining in heart failure patients. Circ Heart Fail. 2018;11(2):e004519. 10.1161/CIRCHEARTFAILURE.117.004519 29382661

[CR12] Horwood CGA, Patel ND, Walker JD, Evans CR. Nonanemic iron deficiency in cardiac surgery: a retrospective observational study. J Cardiothorac Vasc Anesth. 2024;38(9):1899–906. 38942683 10.1053/j.jvca.2024.05.039

[CR13] Hung KC, Chang LC, Ho CN, Hsu CW, Yu CH, Wu JY, et al. Efficacy of intravenous iron supplementation in reducing transfusion risk following cardiac surgery: an updated meta-analysis of randomised controlled trials. Br J Anaesth. 2024;133(6):1137–49. 39332997 10.1016/j.bja.2024.08.030

[CR14] Jain R, Sachdev S, Marwaha N, Gupta A. Assessment of hemoglobin content of packed red cells: a prospective study on hemoglobin content variation due to donor, collection, and processing-related factors. Is it time to label each unit with hemoglobin content? Global Journal of Transfusion Medicine. 2022;7(1):36.

[CR15] Kim HB, Shim JK, Ko SH, Kim HR, Lee CH, Kwak YL. Effect of iron deficiency without anaemia on days alive and out of hospital in patients undergoing valvular heart surgery. Anaesthesia. 2022;77(5):562–9. 35262180 10.1111/anae.15681

[CR16] Martens P, Augusto SN, Mullens W, Tang WHW. Meta-analysis and metaregression of the treatment effect of intravenous iron in iron-deficient heart failure. JACC Heart Fail. 2023;S2213–1779(23):00757–66.

[CR17] Meybohm P, Schnitzbauer A, Bechstein WO, Schmitz-Rixen T, Marzi I, Lustenberger T, et al. Automatized OPS-based calculation of likelihood of RBC concentrate transfusion in a hospital. Anästh Intensiv. 2020;61:140–53.

[CR18] Meybohm P, Schmitt E, Choorapoikayil S, Hof L, Old O, Müller MM, et al. German Patient Blood Management Network: effectiveness and safety analysis in 1.2 million patients. Br J Anaesth. 2023;131(3):472–81. 10.1016/j.bja.2023.05.006 37380568

[CR19] Miles LF, Pac Soo V, Braat S, Bade-Boon J, Heritier S, Klein AA, et al. Associations between non-anaemic iron deficiency and outcomes following elective cardiac surgery (IDOCS): a prospective cohort study. Lancet Haematol. 2022J;9(7):e514–22. 35772430 10.1016/S2352-3026(22)00142-9

[CR20] Muñoz M, Gómez-Ramírez S, Campos A, Ruiz J, Liumbruno GM. Pre-operative anaemia: prevalence, consequences and approaches to management. Blood Transfus. 2015July;13(3):370–9. 26192787 10.2450/2015.0014-15 PMC4614288

[CR21] Muñoz M, Acheson AG, Auerbach M, Besser M, Habler O, Kehlet H, et al. International consensus statement on the peri-operative management of anaemia and iron deficiency. Anaesthesia. 2017;72(2):233–47. 27996086 10.1111/anae.13773

[CR22] Murphy CJ, Oudit GY. Iron-overload cardiomyopathy: pathophysiology, diagnosis, and treatment. J Card Fail. 2010;16(11):888–900. 21055653 10.1016/j.cardfail.2010.05.009

[CR23] Peri V, Devlin P, Perry L, Richards T, Miles LF. Associations between nonanemic iron deficiency and postoperative outcomes in cardiac surgery: a systematic review and meta-analysis. Anesth Analg. 2024;139(1):47–57. 38241670 10.1213/ANE.0000000000006822

[CR24] Piednoir P, Allou N, Driss F, Longrois D, Philip I, Beaumont C, et al. Preoperative iron deficiency increases transfusion requirements and fatigue in cardiac surgery patients: a prospective observational study. Eur J Anaesthesiol. 2011;28(11):796–801. 21885979 10.1097/EJA.0b013e32834ad97b

[CR25] Rössler J, Schoenrath F, Seifert B, Kaserer A, Spahn GH, Falk V, et al. Iron deficiency is associated with higher mortality in patients undergoing cardiac surgery: a prospective study. Br J Anaesth. 2020;124(1):25–34. 31668348 10.1016/j.bja.2019.09.016

[CR26] Song JW, Soh S, Shim JK, Lee S, Lee SH, Kim HB, et al. Effect of perioperative intravenous iron supplementation for complex cardiac surgery on transfusion requirements: a randomized, double-blinded placebo-controlled trial. Ann Surg. 2022;275(2):232–9. 34171864 10.1097/SLA.0000000000005011

[CR27] Spahn DR, Schoenrath F, Spahn GH, Seifert B, Stein P, Theusinger OM, et al. Effect of ultra-short-term treatment of patients with iron deficiency or anaemia undergoing cardiac surgery: a prospective randomised trial. Lancet. 2019;393(10187):2201–12. 31036337 10.1016/S0140-6736(18)32555-8

[CR28] Triphaus C, Judd L, Glaser P, Goehring MH, Schmitt E, Westphal S, et al. Effectiveness of preoperative iron supplementation in major surgical patients with iron deficiency: a prospective observational study. Ann Surg. 2021;274(3):e212–9. 10.1097/SLA.0000000000003643 31939751

[CR29] van Vessem ME, Palmen M, Couperus LE, Mertens B, Berendsen RR, Tops LF, et al. Incidence and predictors of vasoplegia after heart failure surgery. Eur J Cardio-Thorac Surg off J Eur Assoc Cardio-Thorac Surg. 2017;51(3):532–8. 10.1093/ejcts/ezw316 28364440

[CR30] Wang CY, Babitt JL. Hepcidin regulation in the anemia of inflammation. Curr Opin Hematol. 2016;23(3):189–97. 26886082 10.1097/MOH.0000000000000236 PMC4993159

[CR31] WHO guideline on use of ferritin concentrations to assess iron status in individuals and populations [Internet]. Geneva: World Health Organization; 2020. (WHO Guidelines approved by the Guidelines Review Committee). Available from: http://www.ncbi.nlm.nih.gov/books/NBK569880/. Cited 2024 Oct 27. 33909381

[CR32] Wittkamp C, Traeger L, Ellermann I, Eveslage M, Steinbicker AU. Hepcidin as a potential predictor for preoperative anemia treatment with intravenous iron-a retrospective pilot study. PLoS ONE. 2018;13(8):e0201153. 30089125 10.1371/journal.pone.0201153 PMC6082514

