# Supplementary material for: Effect of (short-term) intravenous iron supplementation in iron-deficient non-anaemic cardiac surgical patients on perioperative outcome
Source: Perioper Med (Lond). 2025 Oct 13;14:107. doi: 10.1186/s13741-025-00596-8 (PMC12516845; doi:10.1186/s13741-025-00596-8)
Supplement: Supplementary file 1 — Additional file 1: Supplemental Fig. 1. Time of iron supplementation before surgery. Supplemental Fig. 2. Utilisation of red blood cell units. RBC = red blood cell. Supplemental Fig. 3. Utilisation of red blood cell units per patient. RBC = red blood cell. Supplemental Fig. 4. Postoperative mechanical ventilation. Supplemental Table 1. Types of surgery included in analysis. CABG = coronary artery bypass graft. Supplemental Table 2. Utilisation of blood products and haemoglobin values in matched patients. RBC = red blood cell, CABG = coronary artery bypass graft, Hb = haemoglobin, *Hb level before iron supplementation, **Hb level after iron supplementation. Supplemental Table 3. Postoperative outcome in matched patients. LOS = length of stay, ICU = intensive care unit, LVEF = left ventricular ejection fraction. Supplemental Table 4. Laboratory profile in matched patients. eGFR = estimated glomerular filtration rate, CKD-EPI = chronic kidney disease epidemiology collaboration, Hb = haemoglobin, ICU = intensive care unit, *first measured value after surgery. Supplemental Table 5. Postoperative outcome in patients with ferritin < 30 µg/l. RBC = red blood cell, Hb = haemoglobin, Pat. = patient, CABG = coronary artery bypass graft, *Hb level before iron supplementation. [file 13741_2025_596_MOESM1_ESM.docx]

# Supplemental Figure 1: Time of iron supplementation before surgery


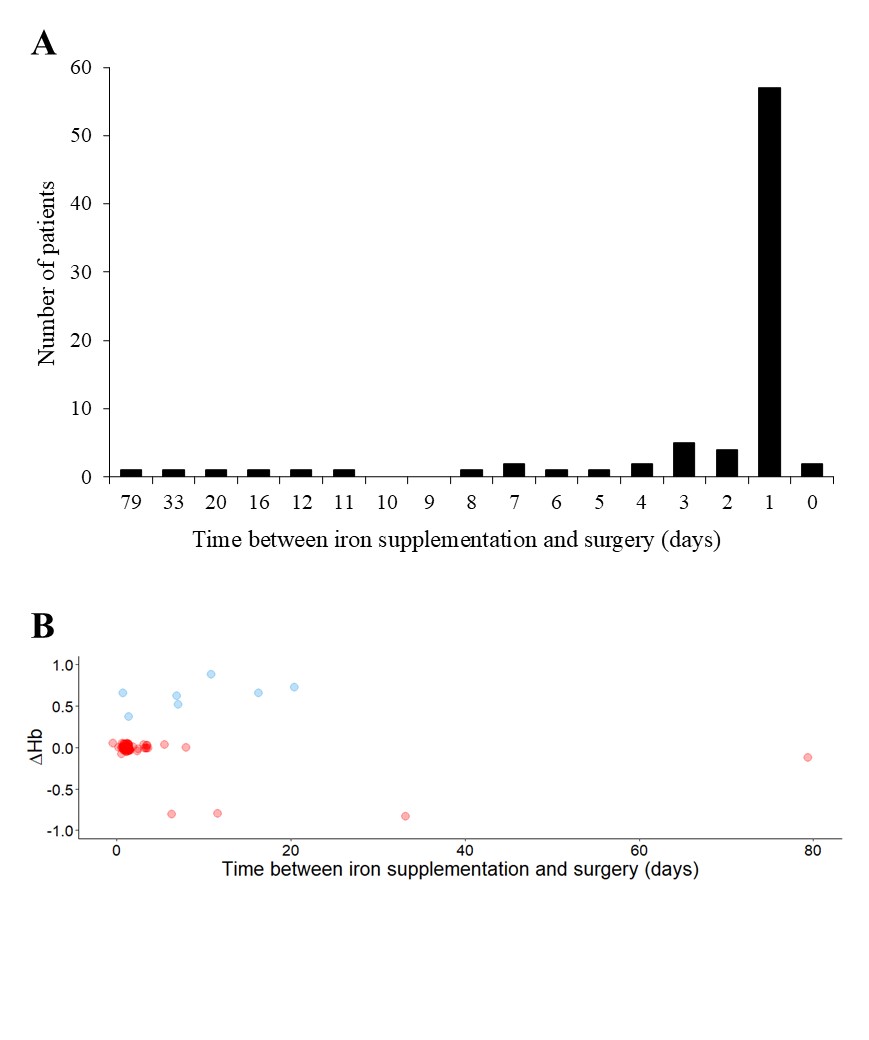


# Supplemental Figure 2: Utilization of red blood cell units


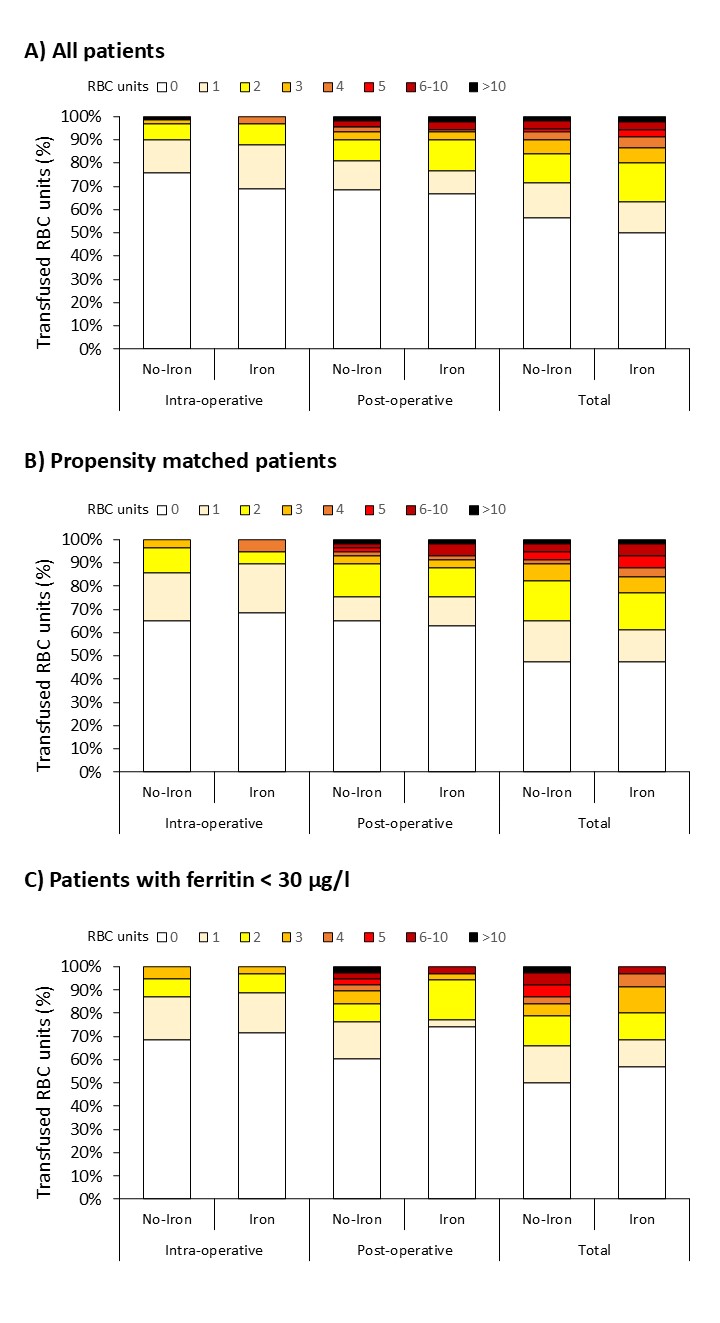


RBC=red blood cell

# Supplemental Figure 3: Utilization of red blood cell units per patient


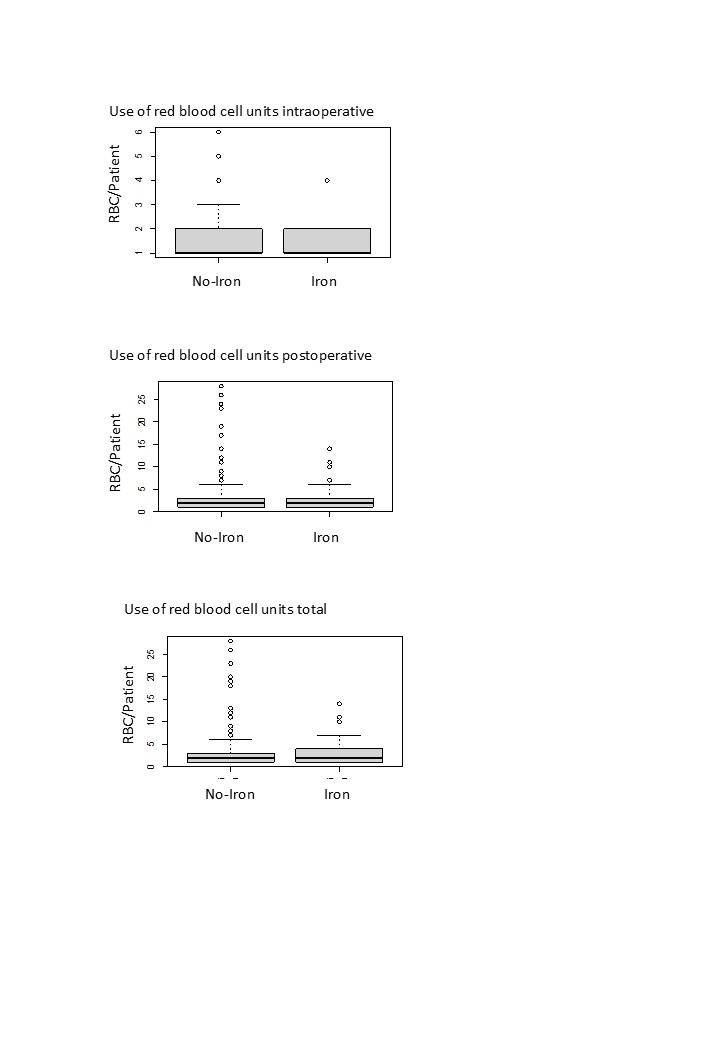


RBC= red blood cell

# Supplemental Figure 4: Postoperative mechanical ventilation


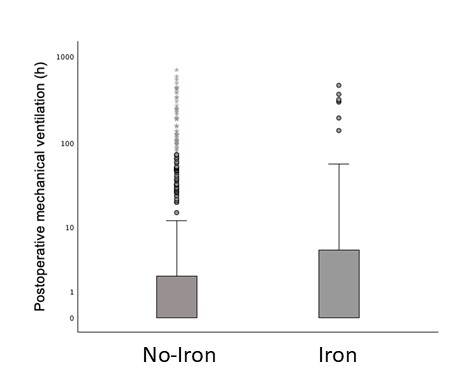


# Supplemental Table 1: Types of surgery included in analysis

|  | **No-Iron**  **N=608** | **Iron**  **N=90** |
| --- | --- | --- |
| CABG + Valve | 45 (7%) | 11 (12%) |
| Valve | 218 (36%) | 34 (38%) |
| Multivalve | 22 (4%) | 4 (5%) |
| Aortic | 37 (6%) | 3 (3%) |
| CABG | 274 (45%) | 37 (41%) |
| Miscellaneous | 12 (2%) | 1 (1%) |

CABG=coronary artery bypass graft

# Supplemental Table 2: Utilization of blood products and hemoglobin values in matched patients

|  | **No-Iron**  **N=57** | **Iron**  **N=57** | **P-value** |
| --- | --- | --- | --- |
| Hb after surgery (g/dl) | 9.7 (95%CI: 9.3-10.1) | 9.9 (95%CI: 9.6-10.3) | 0.20 |
| Hb at discharge (g/dl) | 10.1 (95%CI: 9.7-10.4) | 10.0 (95%CI: 9.6-10.4) | 0.98 |
| RBC transfusion rate (%) |  |  |  |
| Total | 52.6% (95%CI: 39.1-65.8) | 52.6% (95%CI: 39.1-65.8) | 1.0 |
| Intraoperative | 35.1% (95%CI: 23.3-48.9) | 31.6% (95%CI: 20.3-45.4) | 0.84 |
| Postoperative | 35.1% (95%CI: 23.3-48.9) | 36.8% (95%CI: 24.8-50.7) | 1.0 |
| RBC/Pat. total | 1 (IQR: 0; 2) | 1 (IQR: 0; 2) | 0.75 |
| RBC/Pat. intraoperative | 0 (IQR: 0; 1) | 0 (IQR: 0; 1) | 0.68 |
| RBC/Pat. postoperative | 0 (IQR: 0; 1) | 0 (IQR: 0; 1) | 0.84 |
| Use of cell salvage | 24.6% (95%CI: 15.3-38.0) | 19.3% (95%CI: 10.5-32.3) | 0.65 |
| Retransfusion^#^ (ml) | 431.2 (95%CI: 204.1-658.3) | 685.9 (95%CI: 353.3-1017.9) | 0.13 |
| CPB -retransfusion (ml) | 545.7 (95%CI: 442.5-649.0) | 500.0 ((95%CI: 441-558.2) | 0.77 |

RBC=red blood cell, CPG=cardiopulmonary bypass, Hb=haemoglobin, *Hb level before iron supplementation, **Hb level after iron supplementation, ^#^call salvaged blood

# Supplemental Table 3: Postoperative outcome in matched patients

|  | **No-Iron**  **N=57** | **Iron**  **N=57** | **P-value** |
| --- | --- | --- | --- |
| Hospital LOS (days) | 12.8 (95%CI: 10.4-15.3) | 12.4 (95%CI: 9.9-14.9) | 0.49 |
| ICU LOS (days) | 2.4 (95%CI: 1.4-3.3) | 3.1 (95%CI: 1.9-4.4) | 0.11 |
| Mortality by any cause | 0 | 5.3% (95%CI: 1.4-15.6) | 0.61 |
| **Complications** | | | |
| LVEF postoperative | 54.7% (95%CI: 52.3-57.0) | 55.2% (95%CI: 51.7-58.7) | 0.24 |
| ≤40 | 8.8% (95%CI: 3.3-20.0) | 17.5% (95%CI: 9.2-30.4) | 0.27 |
| 41-49 | 14.0% (95%CI: 6.7-26.4) | 15.8% (95%CI: 7.9-28.4) | 1.0 |
| ≥50 | 77.2% (95%CI: 63.8-86.8) | 66.7% (95%CI: 52.8-78.3) | 0.30 |
| Dialysis | 5.3% (95%CI: 1.4-15.6) | 8.8.% (95%CI: 3.3-20.0) | 0.71 |
| Cerebral ischemic event | 0 | 0 | 0 |
| Antibiotic therapy | 15.8% (95%CI: 7.9-28.4) | 19.3% (95%CI: 10.5-32.3) | 0.81 |
| Resurgery | 8.8% (95%CI: 3.3-20.0) | 15.8% (95%CI: 7.9-28.4) | 0.39 |
| Sepsis | 1.8% (95%CI: 0.1-10.6) | 3.6% (95%CI: 0.6-13.2) | 1.0 |
| Duration of mechanical ventilation (h) | 28.5 (95%CI: 5.9-51.1) | 38.1 (95%CI: 12.3-63.9) | 0.10 |

LOS=length of stay, ICU=intensive care unit, LVEF=left ventricular ejection fraction

# Supplemental Table 4: Laboratory profile in matched patients

|  | **No-Iron**  **N=57** | **Iron**  **N=57** | **p-Value** |
| --- | --- | --- | --- |
| **Preoperative period** | | | |
| Creatinine (mg/dl) | 0.9 (95% CI: 0.9-1.0) | 1.0 (95% CI: 0.9-1.1) | 0.54 |
| eGFR to CKD-EPI (ml/min/1.73 m^2^) | 77.0 (95% CI: 71.3-82.7) | 73.6 (95% CI: 68.4-78.8) | 0.61 |
| Urea (mg/dl) | 38.6 (95% CI: 34.4-42.7) | 36.9 (95% CI: 33.3-40.5) | 0.60 |
| Interleukin-6 (pg/ml) | 5.7 (95% CI: 4.2-7.1) | 5.5 (95% CI: 4.2-6.9) | 0.83 |
| **Postoperative period*** | | | |
| Phosphate (mg/dl) | 3.5 (95% CI: 3.3-3.8) | 3.4 (95% CI: 3.2-3.7) | 0.29 |
| Creatinine (mg/dl) | 0.9 (95% CI: 0.9-1.0) | 1.0 (95% CI: 0.9-1.1) | 0.54 |
| eGFR to CKD-EPI (ml/min/1,73 m^2^) | 77.0 (95% CI: 71.3-82.7) | 75.0 (95% CI: 69.6-80.4) | 0.61 |
| Urea (mg/dl) | 32.3 (95% CI: 28.9-35.6) | 33.0 (95% CI: 29.3-36.7) | 0.94 |
| Interleukin-6 (pg/ml) | 626.1 (95% CI: 396.0-856.2) | 1,308 (95% CI: 480.8-2,135.9) | 0.39 |
| **At discharge from ICU** | | | |
| Phosphate (mg/dl) | 3.8 (95% CI: 3.5-4.0) | 3.7 (95% CI: 3.4-4.1) | 0.72 |
| Creatinine (mg/dl) | 1.0 (95% CI: 0.9-1.2) | 1.1 (95% CI: 0.9-1.2) | 0.90 |
| eGFR to CKD-EPI (ml/min/1,73 m^2^) | 73.0 (95% CI: 66.6-79.3) | 74.1 (95% CI: 67.1-81.1) | 0.69 |
| Urea (mg/dl) | 40.7 (95% CI: 34.2-47.3) | 41.2 (95% CI: 35.0-47.3) | 0.97 |
| Interleukin-6 (pg/ml) | 149.9 (95% CI: 96.1-203.7) | 376.2 (95% CI: 52.6-699.9) | 0.83 |

eGFR=estimated glomerular filtration rate, CKD-EPI= chronic kidney disease epidemiology collaboration, Hb=haemoglobin, ICU=intensive care unit, *first measured value after surgery

# Supplemental Table 5: Postoperative outcome in patients with ferritin < 30 µg/l

|  | **No-Iron**  **N=38** | **Iron**  **N=35** | **P-value** |
| --- | --- | --- | --- |
| Hb at discharge (g/dl) | 9.8 (95% CI: 9.5-10.2) | 10.3 (95% CI: 9.8-10.8) | 0.10 |
| RBC transfusion rate |  |  |  |
| Total | 50.0% (95% CI: 34.9-65.2) | 42.9% (95% CI: 26.8-60.5) | 0.71 |
| Intraoperative (%) | 31.6% (95% CI: 18.0-48.8) | 28.6% (95% CI: 15.2-46.5) | 0.98 |
| Postoperative (%) | 39.5% (95% CI: 24.5-56.6) | 25.7% (95% CI: 13.1-43.6) | 0.32 |
| RBC/Pat. total (units) | 0.5 (IQR: 0.0; 2.0) | 0.0 (IQR: 0.0; 2.0) | 0.59 |
| RBC/Pat. intraoperative (units) | 0.0 (IQR: 0.0; 1.0) | 0.0 (IQR: 0.0; 1.0) | 0.56 |
| RBC/Pat. postoperative (units) | 0.0 (IQR: 0.0; 1.0) | 0.0 (IQR: 0.0; 1.0) | 0.27 |
| Use of cell salvage | 26.3% (95%CI: 14.0-43.4) | 25.7% (95%CI: 13.1-43.6) | 1.00 |
| Retransfusion (ml)^#^ | 449.6 (95% CI: 134.0-765.2) | 570.1 (95% CI: 290.2-850.0) | 0.33 |
| CPG (ml) | 505.0 (95% CI: 388.9-621.1) | 447.5 (95% CI: 393.0-502.0) | 0.87 |

RBC=red blood cell, Hb=haemoglobin, Pat.=patient, CPG=cardiopulmonary bypass, *Hb level before iron supplementation, ^#^cell salvaged blood
